# Two competitive nucleation mechanisms of calcium carbonate biomineralization in response to surface functionality in low calcium ion concentration solution

**DOI:** 10.1093/rb/rbv010

**Published:** 2015-08-17

**Authors:** Hua Deng, Shuo Wang, Xiumei Wang, Chang Du, Xingcan Shen, Yingjun Wang, Fuzhai Cui

**Affiliations:** ^1^State Key Laboratory of New Ceramics and Fine Processing, School of Materials Science and Engineering, Tsinghua University, Beijing 100084, China;; ^2^Department of Chemistry and Biochemistry, Jackson State University, Jackson, MS 39203, USA;; ^3^School of Materials Science and Engineering, South China University of Technology, Guangzhou 510641, China and; ^4^Key Laboratory for the Chemistry and Molecular Engineering of Medicinal Resources, School of Chemistry and Chemical Engineering, Guangxi Normal University, Guilin 541004, China

**Keywords:** calcium carbonate, biomineralization, self-assembled monolayer, prenucleation clusters, ions adsorption

## Abstract

Four self-assembled monolayer surfaces terminated with –COOH, –OH, –NH_2_ and –CH_3_ functional groups are used to direct the biomineralization processes of calcium carbonate (CaCO_3_) in low Ca^2+^ concentration, and the mechanism of nucleation and initial crystallization within 12 h was further explored. On −COOH surface, nucleation occurs mainly via ion aggregation mechanism while prenucleation ions clusters may be also involved. On −OH and −NH_2_ surfaces, however, nucleation forms via calcium carbonate clusters, which aggregate in solution and then are adsorbed onto surfaces following with nucleation of amorphous calcium carbonate (ACC). Furthermore, strongly negative-charged −COOH surface facilitates the direct formation of calcites, and the −OH and −NH_2_ surfaces determine the formation of vaterites with preferred crystalline orientations. Neither ACC nor crystalline CaCO_3_ is observed on −CH_3_ surface. Our findings present a valuable model to understand the CaCO_3_ biomineralization pathway in natural system where functional groups composition plays a determining role during calcium carbonate crystallization.

## Introduction

As the most abundant biomineral in nature [1], calcium carbonate usually has astonishing morphologies and structures, such as calcite single crystals in ophiocomid brittlestars [2] and sea urchin spine [3, 4], vaterite in freshwater lackluster pearls [5], biogenic aragonite in mollusk [6, 7], and amorphous calcium carbonate (ACC) in molt of armadillidium vulgare [8]. What are the underlying nucleation and crystallization mechanisms of their formation? This is the key question to clarify the pathway of CaCO_3_ formation in natural system [9–11].

Two nucleation mechanisms have been successively proposed. The first one is classical nucleation theory that assumes crystals nucleate and grow via ion adsorption [12, 13]. The other one was proposed recently that CaCO_3_ biominerals can be formed via stable prenucleation-stage clusters with aggregation into ACC phase by colliding and coalescing and then transforming to a crystal phase [11]. The precritical clusters were confirmed under a stearic acid monolayer using cryotransmission electron microscopy (TEM) by Pouget *et al.* [10]. These two theories appear to be contradictory and many details remain ambiguous [14, 15]. Notably, the impurity-free picoliter droplet arrays were used to study crystal growth in spatially and chemically controlled, finite-reservoir environments. These confined volumes significantly slow CaCO_3_ crystallization proceeds, facilitating observation of ACC during crystallization progresses [16].

Another key point of CaCO_3_ biomineralization is ACC, from which the crystalline phase is transformed [17, 18]. The pure form of ACC is highly unstable while ACC in organisms usually contains many additives, including polyphosphonates, amino acid, oligosaccharide and propylene glycol [19]. Rodriguez-Blanco *et al.* [20] investigated the kinetics and mechanisms of ACC crystallization to calcite via vaterite. All these findings show a profile of CaCO_3_ biomineralization, whereas many details remain unclear. For instance, what is the determinant of the anchoring location of ACC particles and what is the controlling factor of crystallization from ACC? The additives existing in natural ACC are believed to play a complex role on transformation of ACC into crystalline phase. While how the whole procedure is achieved?

To figure out these details, we prepared a two-dimensional biomimetic surface by self-assembling of alkanethiols on gold [21, 22]. Alkanethiols are terminated with −COOH, −OH, −NH_2_ and −CH_3_, which are the most common components of various natural substances involved in biomineralization, including amino acid [23], protein [24, 25], collagen [26, 27], etc. By imitating these biomaterials at molecular level, it is hoped that CaCO_3_ crystallization on theses biomimetic surfaces can bring us deep insight in CaCO_3_ nucleation and crystallization details in living organisms. Previously, we examined the CaCO_3_ crystallizations on different self-assembled monolayers (SAMs) after 12, 24 and 36^ ^h of incubation in low, medium and high Ca^2+^ concentrations of solutions, which indicated that nucleation and crystallization were completed within 12 h and the crystalline phase, size and shape could be modulated by surface chemistries and calcium ion concentrations [28]. In this study, we therefore investigated the detailed CaCO_3_ nucleation mechanisms and crystallization pathway occurred within 12 h in low Ca^2+^ concentration.

## Materials and Methods

### Biomimetic surfaces

Four types of biomimetic surfaces are prepared by a well-developed technology, alkanethiols self-assembling on gold (111) [23]. Water contact angle measurement, X-ray photoelectron spectroscopy and atomic force microscope were utilized to characterize the self-assembled surfaces, which indicated that the SAMs of alkanethiols terminated with different functional groups had similar packing density, as described in our previous reports [28, 29]. The zeta potentials of different surfaces were measured using SurPass Electrokinetic Analyzer (Anton Paar, Austria) in saline background solution at pH 7.4. Because a monovalent cation solution is necessary for calculation in the system, saline was applied instead of CaCl_2_ solution. Eight experimental points were collected for each surface.

### Experimental set-up

**Figure 1.**
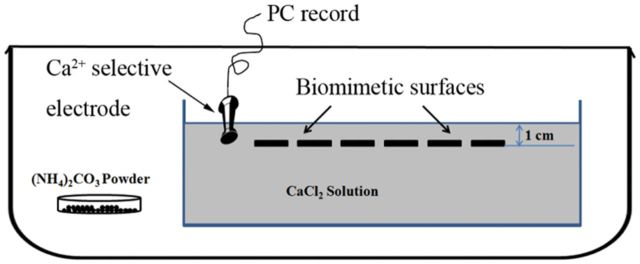
Schematic drawing of experimental equipment. CO_2_ produced by (NH_4_)_2_CO_3_ decomposition diffuses into the solution and reacts with CaCl_2_. The calcium ion selective electrode is placed around the biomimetic surfaces.

The surfaces fixed in a plastic framework were placed upside-down in 1 mM CaCl_2_ solution in a beaker at room temperature. As shown in Fig. 1, the depth was about 1 cm from aqueous surface. The beaker was placed in a closed desiccator containing vials of ammonium carbonate (approximately 5.0 g). The use of (NH_4_)_2_CO_3_ helps to produce CO_2_ and maintain constant pH in the solution [30]. To monitor the concentration of free Ca^2+^ near the biomimetic surfaces, a calcium ion selective electrode (Shanghai Sassin, China) is placed as close as possible to the surfaces and the data are recorded by software named Measurement Program-500 (MP-500, Shanghai Sassin, China). The electrode was calibrated utilizing 0.01 mM and 1 mM CaCl_2_ solution successively before every measurement. To minimize contamination of SAMs, the substrates were placed into CaCl_2_ solution immediately after cleaning with triple-distilled water. Low concentration of CaCl_2_ was utilized here to mimic the biomineralization process in nature as far as possible, which was different with previous reports [30–32]. Our previous study [28] demonstrated that CaCO_3_ crystallization was directed by functional groups in low concentration of Ca^2+^ solution whereas nonspecific precipitation occurred in high Ca^2+^ concentration solution.

### Characterizations on biomineralized surfaces

Biomimetic surfaces were taken out after crystallization for 15 min, 0.5, 1, 3 and 12 h, followed by slight washing for 2–3 times. The surfaces are then immediately placed and dried in nitrogen atmosphere. CaCO_3_ depositions formed on biomimetic surfaces were analyzed *in situ* utilizing X-ray diffraction (XRD), scanning electron microscope (SEM) and attenuated total reflectance of Fourier transform infrared spectroscopy (ATR-FTIR). Notably, exposure of CaCO_3_ sample in air should be as short as possible and characterized straight away because the pure ACC particles are only stable for a short time in specific conditions and inclined to transform to stable crystal phase [10, 33]. XRD was carried out on D/max 2500 (Rigaku, Japan) with Cu Kα1 radiation (λ = 0.1541 nm) at room temperature. The 2θ ranged from 15° to 75° and XRD profile was recorded in step-scan intervals of 0.02° at a scanning speed of 6.0°/min at 40 kV and 200 mA. SEM images were carried out on S-4500 (Hitachi, Japan) with an accelerating voltage of 15 kV. The ATR-FTIR spectra were collected on Spectrum 400 (PE, USA) with the wavelength range 650–4000 cm^−^^1^. The samples were pressed against a Ge prism tip at the focal position to obtain the spectrum, and 16 scans were collected at a resolution of 4 cm^−^^1^.

## Results

### Developments of Ca^2+^ concentration around four types of surfaces

**Figure 2.**
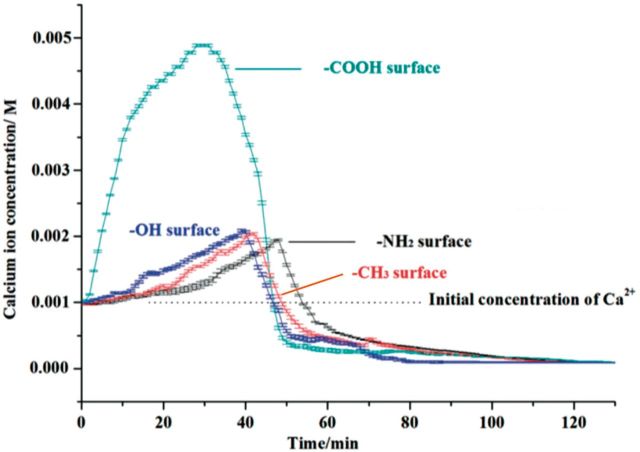
Development of Ca^2+^ concentration over time measured by calcium ion selective electrode. Curves of Ca^2+^ concentration around four functional group surfaces are shown. The data are present as average ± SD.

The changes of free Ca^2+^ concentration around four surfaces are shown in Fig. 2. Two distinct trends are observed. Around −COOH surface, a sharp increase occurs once the surface is immersed into solution. At about 30 min, Ca^2+^ concentration is the highest, approximately five times more than initial concentration. Then, a slow decrease takes place with a growing gradient. The Ca^2+^ concentration reaches the lowest and maintains constant after 50 min. Changes of Ca^2+^ concentration around −OH, −NH_2_ and −CH_3_ surfaces are similar. A slow increase with a growing gradient occurs and was followed by a fast decrease with a reducing gradient. The zeta potential of −COOH and −OH surfaces at pH ∼ 7.4 was −28.3 ± 4.8 and −19.8 ± 1.0 mV, respectively, which indicated that the two types of surfaces generated negative charges through ionization in solution. In contrast, the zeta potential of the −NH_2_ surfaces was 1.4 ± 0.2 mV indicating the positively charged surfaces. Besides, it is noted that the −CH_3_ surface also showed negative zeta potential (−15.2 ± 3.5 mV) at the neutral solution. This suggested that the methyl-terminated surface preferentially adsorbed negative ions because cations are more easily hydrated and retained in solution, which was consistent with previous studies [34]. This is probably why the calcium concentration near the −CH_3_ surfaces was a little bit higher than that near the −NH_2_ surfaces during the first 30 min in Fig. 2.

The initial concentration of free calcium ion is 1 mM, as shown by the black dotted line. Once the −COOH substrates were placed into solution, a sharp increase of [Ca^2+^] occurred within the first 15 min because of the strongly negative-charged surfaces, which implied the free Ca^2+^ in solution were moving toward −COOH surface, symbolizing the quick aggregation of calcium ions via electrostatic adsorption. Whereas only extremely slow increases of [Ca^2+^] near −OH, −NH_2_ and −CH_3_ surfaces were observed initially because the weak negative potentials of −OH and −CH_3_ surfaces comparing with −COOH surface contributed to the slow aggregation of calcium. After that, with carbon dioxide being generated and dissolving into upper solution gradually, calcium ions were consumed to form carbonate and osmosis pressure in upper solution decreased. To balance this change, the calcium ions in middle and bottom of solution moved to upper area, causing a slow increase of calcium ion around substrates after 20 min. Besides, although −NH_2_ surface had a positive potential, the curve of Ca^2+^ changes over time was similar with −OH and −CH_3_ surfaces indicating the carbonate ions formed in upper solution dominated the aggregation of calcium cations instead of the negative charges of the substrate surfaces.

### Nucleation and evolution of CaCO_3_ crystallization on −COOH surface

**Figure 3.**
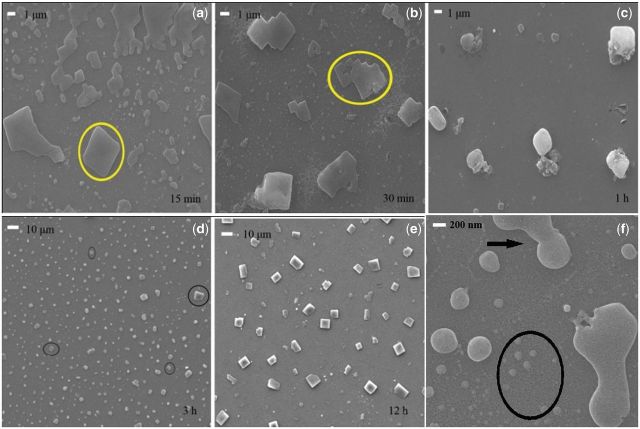
SEM images of CaCO_3_ formed on −COOH surface after (**a**) 15 min, (**b**) 30 min, (**c**) 1 h, (**d**) 3 h and (**e**) 12 h of crystallization. (**f**) High-magnification image of (a). CaCO_3_ nanoparticles covered the surface (black circle in (f)) and grow larger by aggregation until a critical size is reached forming regular edges (circles in (a) and (b)). Spheroidic CaCO_3_ phases are observed and some calcite rhombohedra are formed at 3 h (black circles in (d)). After crystallization for 12 h, oriented calcite rhombohedra are formed.

**Figure 4.**
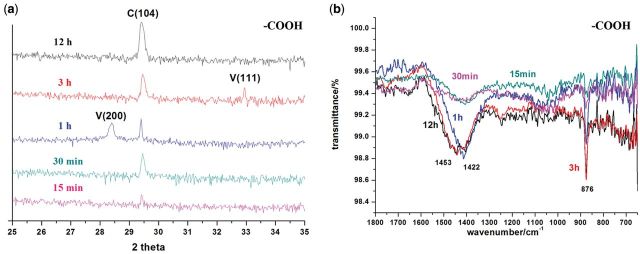
XRD patterns (**a**) and FTIR-ATR spectra (**b**) of CaCO_3_ formed on −COOH surface at 15, 30 min, 1, 3 and 12 h. C, calcite; V, vaterite.

Nucleation and crystallization of CaCO_3_ on −COOH surface after 15, 30 min, 1, 3 and 12 h of reaction were examined by SEM, XRD and FTIR, as shown in Figs. 3 and 4. After 15 min of reaction, CaCO_3_ nanoparticles ranged from tens of nanometers to about 100 nm were observed on −COOH surface, as shown in Fig. 3f. It is very interesting that these small CaCO_3_ nanoparticles integrate together and merge with each other to form the droplet-shaped CaCO_3_ nanoparticles with larger size. The sizes of these CaCO_3_ nanoparticles range from 100 to 800 nm and then seem to flow and grow larger by coalescing, until a critical size is reached, as indicated by black arrows in Fig. 3f. After nucleation, the CaCO_3_ nanoparticles gradually transform into crystals, as shown in the Fig. 3a–e. Considering the sharp increase of Ca^2+^ concentration near the −COOH surface initially, calcium ion adsorption onto −COOH surface should be also occurred in the meanwhile Ca^2+^ and CO_3_^2^^−^ aggregate together forming prenucleation clusters once enough CO_3_^2^^−^ formed in solution. Because it takes time for CO_3_^2^^−^ formation in solution, CaCO_3_ cluster formation was slowed down that makes the classical ions accumulation mechanism reasonable in our case. After that, these clusters can also be adsorbed and gathered onto the strongly negatively charged −COOH surface.

XRD examinations showed that no obvious strong peak was observed in XRD at 15 min, indicating the CaCO_3_ nanoparticles are mostly ACC. Calcite rhombohedra with oriented (104) were formed under the control of −COOH surface while vaterite phase was also observed. In the following several hours, diffraction peaks of (200) and (111) crystal faces of vaterite are detected by XRD, as indicated in Fig. 4a. This is in accordance with the involvement of ACC nanoparticle-based crystallization. Previous studies have shown that ACC nanoparticles formed in solution are adsorbed to the upper layer of calcite formed at initial stage, and finally transformed to calcite via vaterite [3, 10, 35].

This CaCO_3_ crystallization pathway is also confirmed by ATR-FTIR spectra (Fig. 4b). A wide band (ν_s_) at 1415 cm^−^^1^ is collected at 15 min, which is overlapping with the peak at 1475 cm^−^^1^ at 30 min. The intensive band (ν_s_) at 1422 cm^−^^1^ is collected at 1 h that gradually shifts to 1453 cm^−^^1^ after 3 h. Simultaneously, a peak (γ) at 876 cm^−^^1^ is observed with an increasing absorbency over time. Previous reports [36–40] show that double bands at 1475 and 1415 cm^−^^1^ of ACC are attributed to stretching vibrations of carbonate ion while peak at 866 cm^−^^1^ is attributed to bending vibrations of carbonate ion. The typical peaks for calcite and vaterite are listed in Table 1. The wide and overlapping band at 1475 and 1415 cm^−^^1^ indicates existence of ACC until 30 min. The peaks at 1422 and 876 cm^−^^1^ suggest that calcite is gradually formed. Almost identical peaks are collected at 3 and 12 h, suggesting crystallization has almost completed, in accordance with SEM results.

**Table 1.** Typical peaks and attribution of FTIR spectra for calcite and vaterite

Calcite (cm^−1^)	Vaterite (cm^−1^)	Attribution
1421	1421	Asymmetric stretching of CO_3_^2−^ (ν_s_)
1082	1082	Symmetric stretching of CO_3_^2−^ (ν_as_)
876	870	Out-of-plane deformation of CO_3_^2−^ (γ)
713	750	In-plane deformation of CO_3_^2−^ (δ)

### Evolution of CaCO_3_ crystallization on −OH and −NH_2_ surfaces

**Figure 5.**
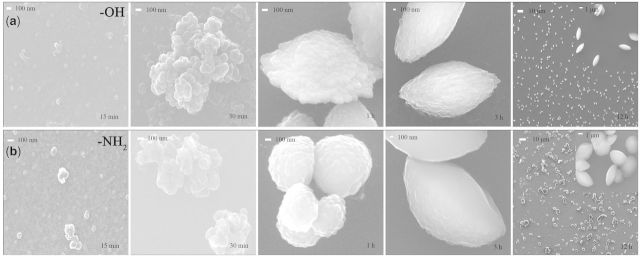
typical SEM images of CaCO_3_ formed on (**a**) −OH and (**b**) −NH_2_ surfaces at 15, 30 min, 1, 3 and 12 h. ACC nanoparticles with 60 ± 20 nm in diameter are adsorbed onto surfaces as nuclei, which are probably formed through aggregation of clusters. Crystallization occurs among ACC particles and propagates orientedly. Spindle-shaped vaterites are formed on −OH surface and spheroidic vaterite on −NH_2_ surface.

**Figure 6.**
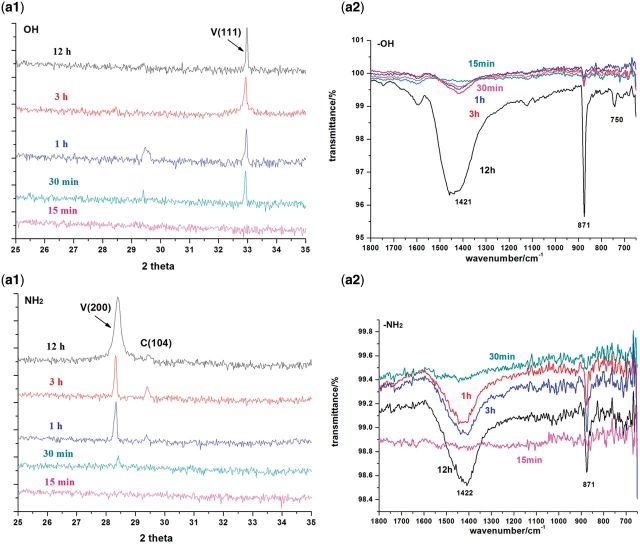
XRD patterns and FTIR-ATR spectra of CaCO_3_ formed on −OH (**a1**, **a2**) and −NH_2_ (**b1**, **b2**) surfaces at 15, 30 min, 1, 3 and 12 h. C, calcite; V, vaterite.

The initial stages of CaCO_3_ crystallization pathways on −OH and −NH_2_ surfaces are almost identical. The typical SEM, XRD and FTIR results are shown in Figs. 5 and 6. At 15 min, no diffraction peak in XRD pattern was observed demonstrating the formation of ACC nanoparticles. SEM images indicated that these ACC nanoparticles were about 60 ± 20 nm that probably formed through aggregation of clusters via prenucleation clusters mechanism [10, 11, 14–16, 41, 42]. The size of particles reached 0.95 ± 0.35 μm after reaction for 30 min. In the following hours, ACC particles continuously aggregate and finally form vaterite. ATR-FTIR spectra of CaCO_3_ formed on biomimetic surfaces also demonstrate a clear pathway for vaterite formation via ACC transformation. As shown in Fig. 5, ACC is formed at initial stage and transformed to vaterite gradually. Oriented (111) and (200) growth of vaterite are observed on −OH and −NH_2_ surfaces, respectively. In ATR-FTIR spectra, the wide band at 1421 cm^−^^1^ is attributed to ν_s_ of ACC whereas the intensive peaks at 1422 and at 871 cm^−^^1^ are typical peaks of vaterite.

Although the −OH surface is negatively charged, the ability of Ca^2+^ adsorption is only a little bit higher than −NH_2_ surface and is much lower than −COOH surface in the first 30 min according to Fig. 2. The similar initial stages of biomineralization on −OH and −NH_2_ surfaces indicated that negative charge of −OH group has little effect on calcium ion absorption and nucleation of calcium carbonate. Although the −CH_3_ substrate generated negative potential, CaCO_3_ crystals formed in solution around the surfaces had no strong affinity with methyl-terminated surfaces. Therefore no nucleation occurs on −CH_3_ surface because of its high hydrophobicity and neutral surface, which has been used as the crystallization-inhibited functional group [43, 44].

## Discussion

In calcium carbonate biomineralization, the nucleation mechanisms still remain debated so far. The key controversial question is the existence conditions of Ca^2+^ ions before nucleation, which are preferentially adsorbed and aggregate onto surfaces or form ions clusters with CO_3_^2^^−^ in solution?

According to our studies, these two pathways should probably exist on −COOH surface competitively dominating the biomineralization process by the winning mechanism. Evidences from Ca^2+^ concentration development and SEM results suggest that the classical nucleation by addition of ions is also involved on −COOH surface. In the early stage of biomineralization, the strongly negative charges of carboxyl groups adsorb calcium ions via electrostatic interactions, which contribute to the instant increase of Ca^2+^ concentration near the −COOH surfaces within the first 30 min. In the area far from the surfaces, the calcium ions could not feel the attractions of negative-charged template, whereas in the area near the surfaces, the Ca^2+^ concentrations are obviously higher than the initial concentration of as-prepared solution because of the aggregations of calcium ions. In this study, the terminated −COOH groups of SAMs we used as template have the closed-packed pattern consisting of the (√3 × √3) R30° structure unit with theoretical area density of 7.68 × 10^−^^6^ mol m^2^ [12]. Therefore, the saturated surface density of calcium ions adsorbed on −COOH surfaces is ∼10^−^^6 ^mol m^2^. According to Fig. 2, the Ca^2+^ concentration near the −COOH surfaces is approximately 4 mM after crystallization for 15 min, indicating the surface density of Ca^2+^ in solution is 7.92 × 10^−^^10 ^mol m^2^ (taken radius of Ca^2+^ as 0.99 Å). Therefore, we could estimate that Ca^2+^ concentration on the −COOH surfaces is much higher than that near the surfaces in low calcium Ca^2+^ concentrations of solutions. In this study, it takes time to accumulate enough CO_3_^2^^−^ in solution for biomineralization. Especially in low calcium ion concentration, localized calcium ions on periodically arranged −COOH groups ion with high surface density would undergo nucleation through ion adsorption mechanism in the early stage. These Ca^2+^ contribute to form oriented crystalline phase, in accordance with oriented (104) calcite from XRD analysis, which has also been reported by Tremel *et al.* [31]*.* Simultaneously, with the accumulation of Ca^2+^ and CO_3_^2^^−^ near the surfaces, electrostatic interaction and Brownian motion of these positive and negative ions facilitate the formation of prenucleation clusters leading to a new pathway for CaCO_3_ biomineralization based on cluster-based mechanism.

**Figure 7.**
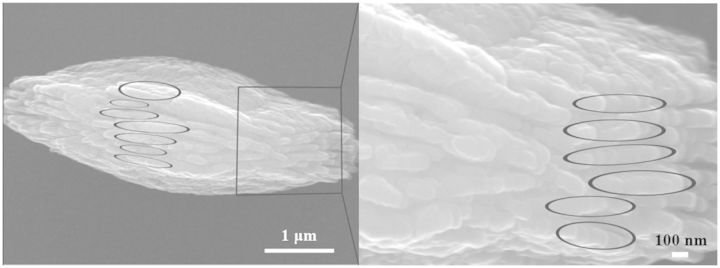
The layer-by-layer growth model of CaCO_3_ on −OH surface. Coalescence of CaCO_3_ nanoparticles in circles demonstrates that crystallization propagates layer-by-layer.

On −OH and −NH_2_ surfaces, the curves of Ca^2+^ concentration development over time are quite different with −COOH group, indicating the week adsorption of Ca^2+^ with the chemical groups of −OH and −NH_2_. Therefore, the CaCO_3_ clusters formed near the surfaces via Brownian motion and electrostatic interaction of Ca^2+^ and CO_3_^2^^−^ in solution will dominate the biomineralization process. Furthermore, crystalline propagation of CaCO_3_ on −OH and −NH_2_ groups was also observed. In our study, crystalline domains begin to develop on −OH and −NH_2_ surfaces once the CaCO_3_ nanoparticles are absorbed onto biomimetic surfaces. Ions at the interface rearrange to form crystalline domain due to the interaction with functional groups of the biomimetic surface. This will guide the rearrangement of the neighboring ions so that crystallization can ‘transmit’. In another word, along with the growth of CaCO_3_ nanoparticles, crystalline domains propagate gradually via oriented secondary nucleation within CaCO_3_ nanoparticles [9]. The involved mechanism may be also hierarchical self-assembling or dissolution and reprecipitation [24, 45–48]. Figure 7 shows the layer-by-layer model for coalescence of CaCO_3_ nanoparticles and propagation of crystalline phase, as the traces of hierarchical self-assembling mechanism. The thermodynamic cue may be that specific plane of crystalline CaCO_3_ phase is favored because the free-energy barrier is decreased owing to the properties of functional groups, i.e. wettability, polarity, electric charge and pattern. This has been extensively discussed by Tremel *et al.* [31, 49] and by Aizenberg *et al.* [30, 42]. These procedures suggest that crystallization originates from the biomimetic surface and spreads from internal to external space of CaCO_3_ particles directed by functional groups.

**Figure 8.**
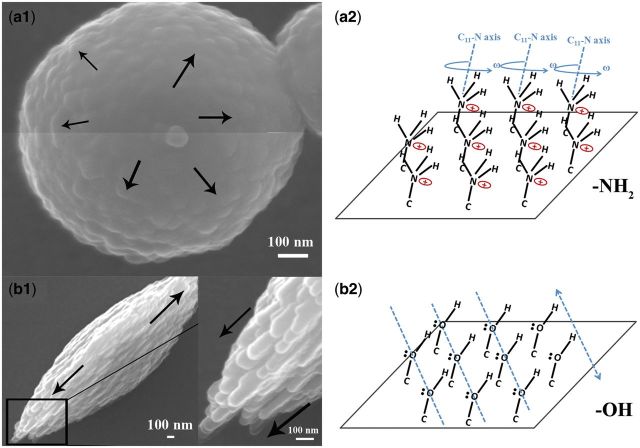
propagation of crystallization via secondary nucleation among CaCO_3_ nanoparticles. SEM morphologies of spheroidic vaterite on −NH_2_ surface (**a1**) and spindle-shaped vaterites on −OH surface (**b1**). (**a2**, **b2**) Geometry of headgroups of −NH_2_ and −OH surfaces. −NH_2_ is free to rotate around C_11_-N axis, as presented by ω. Rotation around C_11_-N axis of −OH group has no effect on the order of the outer part of the surface. Hydrogen atom can interact with neighboring oxygen atom to form hydrogen bond. The dotted line in (b2) presents the arranged oxygen atoms in line and the arrow presents the direction of the second nucleation among CaCO_3_ nanoparticles.

After reaction for about 1 h, vaterites formed on −OH and −NH_2_ surfaces gradually exhibit distinct morphologies and orientation, which is caused by secondary nucleation among CaCO_3_ nanoparticles (Fig. 8). On −OH surface, CaCO_3_ nanoparticles nucleate toward two opposite directions and form spindle-shaped vaterite at last and orientation of (111) is preferred. On −NH_2_ surface, however, no oriented nucleation of CaCO_3_ nanoparticles is observed and crystallization spreads around. As a result, spheroidic vaterite is formed with favored (200) orientation. This can be explained by the ambiguity in the precise orientation of the headgroups and uncertainty in the order of the outer part of the SAM [30]. The terminal groups are free to rotate around the C*n*-X bond, and are capable of interacting with each other. As shown in Fig. 8, for −NH_2_ terminated surface, rotation ω leads to uncertainty in the order of the outer part of the surface. In consequence, direction of secondary nucleation among CaCO_3_ nanoparticles is uncertain. However, the order of the outer part of the −OH surface is fixed owing to the hydrogen bond. The secondary nucleation among CaCO_3_ nanoparticles takes place toward two opposite directions.

Weiner and Addadi [9] have introduced a hypothetical pathway in a sea urchin spicule about how the crystallization propagates by secondary nucleation among ACC particles. In our study, TEM images provide direct evidences that vaterites with different morphologies and orientations are formed via the oriented secondary nucleation among CaCO_3_ nanoparticles. CaCO_3_ nanoparticles formed on −OH surfaces have the size ranged from 60 to 80 nm, extremely similar with that in a sea urchin spicule. Morphologies and shapes of CaCO_3_ nanoparticles suggest the directed and oriented coalescences, i.e. the oriented secondary nucleation of CaCO_3_ nanoparticles functions as a key strategy of vaterite formation.

## Conclusion

The present study presents a clear CaCO_3_ biomineralization pathway by introducing biomimetic surfaces terminated with four types of common functional groups in nature. Ion adsorption mechanism is proved to be reasonable on strongly negative-charged −COOH surface in low calcium condition. In most instances, biomineralization occurs via stable prenucleation clusters. In natural system, various functional groups should play a compositive role on CaCO_3_ crystallization and the electrostatic interaction of −COOH surface may be eliminated due to the low percentage of composition and the irregular arrangement of −COOH groups. As a result, CaCO_3_ nucleation tends to be cluster-based and crystallization propagates mainly via secondary nucleation among ACC nanoparticles, which is similar with that on −OH and −NH_2_ surfaces. Also, our findings supply direct evidence for the secondary nucleation among ACC particles by which the crystallization propagates in natural system. The functional groups play a fundamental but complicated role on morphology and orientation of crystals. Our findings will be helpful for understanding and mimicking the role of various natural biomaterials on CaCO_3_ biomineralization from molecular level.
